# T肽增强顺铂肿瘤杀伤作用的研究及机制探讨

**DOI:** 10.3779/j.issn.1009-3419.2017.02.01

**Published:** 2017-02-20

**Authors:** 宏毅 张, 明辉 刘, 颖 李, 永文 李, 嵩 徐, 振华 潘, 明彪 李, 海洋 范, 红雨 刘, 军 陈

**Affiliations:** 1 300052 天津，天津医科大学总医院肺部肿瘤外科 Department of Lung Cancer Surgery; 2 天津市肺癌研究所，天津市肺癌转移与肿瘤微环境实验室 Tianjin Key Laboratory of Lung Cancer Metastasis and Tumor Microenvironment, Tianjin Lung Cancer Institute, Tianjin Medical University General Hospital, Tianjin 300052, China

**Keywords:** 肺肿瘤, T肽, TNF-α, IFN-γ, Lung neoplasms, T peptide, TNF-α, IFN-γ

## Abstract

**背景与目的:**

T肽是Tuftsin的衍生物，其基本功能是刺激巨噬细胞，提高巨噬细胞吞噬及分泌能力，通过增强效应细胞的细胞杀伤作用而被广泛用于多种恶性肿瘤术后抗肿瘤治疗。本研究旨在探讨T肽与化疗药物顺铂的联合能否提高铂类药物的抗肿瘤疗效及机制。

**方法:**

应用酶联免疫吸附剂测定（enzyme-linked immunosorbent assay, ELISA）T肽和/或顺铂处理人巨噬细胞株U937后肿瘤坏死因子-α（tumor necrosis factor-α, TNF-α）和干扰素-γ（interferon-γ, IFN-γ）分泌水平的变化；应用裸鼠荷瘤动物模型，分析T肽和/或顺铂处理小鼠肿瘤变化情况以及小鼠用药期间外周血中巨噬细胞变化情况。

**结果:**

①应用ELISA检测发现在T肽组和T肽联合顺铂组中，U937细胞培养液中TNF-α水平明显高于对照组和顺铂组；T肽联合顺铂组中IFN-γ水平明显高于对照组、顺铂组和T肽组；②在小鼠荷瘤模型中，T肽联合顺铂组的肿瘤体积明显小于对照组、顺铂组和T肽组，并且小鼠没有明显的体重下降；③在小鼠外周血的检测分析中发现，T肽联合顺铂组中活化巨噬细胞数呈现增长趋势。免疫组化分析各组中肿瘤组织Ki67显示T肽联合顺铂组抑制肿瘤细胞增殖。

**结论:**

T肽能够促进巨噬细胞增殖和促进其分泌肿瘤细胞杀伤因子（TNF-α、IFN-γ）；在体内动物模型中，T肽能够提高化疗药物如顺铂的疗效，使联合组具有更强的肿瘤抑制作用，同时能够减轻化疗药物的毒副作用。

## Abstract (en)

**Background and objective:**

T peptide is extensively used in anti-tumor treatment. The aims of this study were to investigate whether T peptide enhances cisplatinum efficiency while reducing its side effects and to identify its effective mechanisms.

**Methods:**

(1) Human macrophage U937 cells were treated with T peptide and/or cisplatinum. The levels of tumor necrosis factor-α (TNF-α) and interferon-γ (IFN-γ) of each group were detected by enzyme-linked immunosorbent assay (ELISA); (2) Xenograft mouse models of human lung cancer were treated with T peptide and/or cisplatinum once every five days for three times. Tumor volumes were measured during treatment; (3) The percentages of macrophages in the peripheral blood of the xenograft mouse models were measured by FACS.

**Results:**

(1) Compared with other groups, the level of TNF-α was significantly higher in the human macrophage U937 cells that were treated with T peptide combined with cisplatinum. The levels of IFN-γ were significantly higher in human macrophage U937 cells that were treated with T peptide alone or T peptide combined with cisplatinum; (2) In the xenograft mouse models, T peptide combined with cisplatinum treatment significantly inhibited tumor growth without weight loss compared with the other groups; (3) The percentages of macrophages in the peripheral blood were significantly higher in the xenograft mouse models that were treated with T peptide combined with cisplatinum compared with in the other groups.

**Conclusion:**

T peptide promotes macrophage proliferation and increases tumor cell killing factors (TNF-α, IFN-γ) *in vitro*. Moreover, T peptide enhances the efficacy of cisplatin and reduces its toxicity *in vivo*.

肿瘤治疗包括手术、化疗、放疗、靶向治疗以及生物治疗等。化疗能抑制恶性肿瘤的生长和发展，并在一定程度上杀死肿瘤细胞。但是，目前常用的化疗药物均缺乏特异性，往往在抑制肿瘤的同时对机体增殖旺盛的细胞，如骨髓细胞、肠上皮细胞、生殖细胞及中枢神经系统细胞有一定的影响；有些药物还对肝、肾、心功能有损伤，少数药物还对皮肤及其附件、肺、内分泌系统有不同程度的损伤；此外，多数抗肿瘤药物都有免疫抑制作用，有潜在的致畸和致癌作用。

Tuftsin是IgG分子Fc段降解产生的由四个氨基酸组成的一段生理肽（Thr-Lys-Pro-Arg, TKPP）^[1]^，被人们认知及研究已有40多年，文献^[2, 3]^证实Tuftsin在体内具有显著的抗肿瘤活性，可以通过巨噬细胞广泛作用于人体的免疫系统，而且毒性较低。但是合成的Tuftsin一直难以应用于临床。首先，合成的Tuftsin由于蛋白酶的水解作用，其体内半衰期仅有0.27 h，进入体内后很快被水解，难以发挥作用。因此阻碍了其在抗肿瘤领域的应用。T肽是Tuftsin的衍生物，与Tuftsin具有相似的生物学功能，但在药代动力学上优于Tuftsin。其化学结构为（TKPR），分子量2, 516。研究^[4]^表明，T肽表现出了比较明显的肿瘤抑制作用和较小的毒副作用。目前，T肽广泛用于多种恶性肿瘤术后抗肿瘤治疗，主要是通过提高肿瘤患者自身免疫力而达到抗肿瘤的作用。

铂类化疗药广泛用于各类肿瘤的治疗中，主要是通过破坏DNA的复制抑制细胞增殖。由于铂类药物对DNA合成的破坏作用是非特异性的，因此在破坏肿瘤细胞复制的同时会对增殖活跃的骨髓干细胞有一定抑制作用。基于T肽具有促进巨噬细胞分化增殖的作用，本研究探讨T肽能否与铂类化疗药物产生协同作用，增强化疗药的抗肿瘤作用，减轻化疗药物的毒副作用，并对其机制进行初步探讨。

## 1 材料与方法

### 1.1 细胞培养和材料

人U937巨噬细胞株购于中科院细胞库，培养于10 cm培养皿，37 ℃、5%CO_2_饱和湿度的培养箱中，培养基为含10%胎牛血清的RPMI 1640培养基。T肽，白色冻干粉末，纯度 > 98%、0.9%NaCl稀释至1 mg/mL，分装成200 μL后储蓄在-20 ℃冰箱备用。顺铂白色粉末，用0.9%NaCl稀释后分装备用。肿瘤坏死因子-α（tumor necrosis factor-α, TNF-α）和干扰素-γ（interferon-γ, IFN-γ）的酶联免疫吸附剂测定（enzyme-linked immunosorbent assay, ELISA）检测试剂盒购于上海博谷公司。CD14、F4/80抗体均购于BD公司。

### 1.2 T肽对巨噬细胞细胞因子分泌作用的研究

U937巨噬细胞常规培养、传代后进行实验。实验分为4组：顺铂组以5 μg/mL处理细胞，T肽组以浓度为400 μmol T肽处理细胞，以及T肽+顺铂组（T肽400 μmol，顺铂5 μg/mL），对照组为0.9%NaCl。处理72 h后收集细胞上清液，应用ELISA方法检测不同处理组细胞因子TNF-α和IFN-γ的变化情况。ELISA实验按照说明书步骤进行。

### 1.3 肺癌移植瘤模型探讨T肽联合顺铂对肿瘤生长的作用

人鳞癌小鼠模型的建立及分组：新鲜人肺鳞癌组织，剪碎，接种于1只-3只裸鼠右侧后肢皮下，使其成瘤。3周后，在无菌条件下分离出肿瘤组织。打碎肿瘤组织后植入40只小鼠左前肢皮下。肿瘤体积达到200 mm^3^-300 mm^3^时入组。分组：A组（T肽）、B组（T肽+顺铂）、C组（顺铂）、D组（control）。药物：实验期间每隔5天给药一次，共给药3次。浓度、给药途径及用药剂量见表 1。本实验数据处理以《细胞毒类抗肿瘤药物非临床研究技术指导原则》和《抗肿瘤药效学指导原则讨论稿》为指导进行数据处理。肿瘤体积计算：V=L^*^W^2^/2（V=肿瘤体积，L=长径，W=短径）；肿瘤生长抑制率（瘤重）=（空白对照组瘤重-给药组瘤重）/空白对照组瘤重^*^100%，肿瘤生长抑制率（体积）=（空白对照组体积-给药组体积）/空白对照组体积^*^100%^[1]^。

### 1.4 流式细胞技术（fluorescence activated sorter, FACs）检测小鼠血中巨噬细胞变化

毛细管蘸抗凝剂取鼠尾血20 μL，加入含500 μL PBS的EP管中，混悬细胞，防止凝集。1, 500 rpm离心4 min，去上清。每管加入300 μL红细胞裂解液，轻轻吹打混匀，裂解10 min。加入500 μL PBS，混匀，1, 500 rpm，4 ℃离心4 min，弃红色上清。加入100 μL Binding buffer重悬细胞沉淀，加入CD14-FITC及F4/80-PE抗体各1 μg，避光孵育20 min，1 500 rpm离心4 min，加入300 μL PBS重悬，上机检测。

### 1.5 免疫组化检测小鼠瘤块中Ki67表达

处死小鼠后无菌条件下分离瘤块，常规石蜡包埋切片，常规切片，用兔抗鼠Ki67单克隆抗体，采用ABC法、DAB显色，苏木精复染。常规光镜检测Ki67的表达情况。以细胞核出现棕黄色颗粒为阳性，随机读取10个高倍视野，共计数1, 000个细胞，以Ki67阳性细胞所占百分数作Ki67指数，以评估Ki67表达水平。染色后显微镜下观察照相。Ki67阳性表达为细胞胞核上呈现棕黄色颗粒；分别对镜下阳性细胞的百分比和染色强度给予评分。阳性着色细胞数：阳性细胞数 < 5%为0分，5%-25%为1分，26%-50%为2分，51%-75%为3分，76%-100%为4分。阳性着色强度：无色为0分，淡黄色为1分，棕黄色为2分，棕褐色为3分。两者计分相乘即为阳性等级：0分为阴性（-），1分-4分为弱阳性（+），5分-8分为阳性（++），9分-12分为强阳性（+++）^[5-7]^。

### 1.6 统计学方法

采用SPSS 13.0统计分析软件和Excel软件进行统计学分析，组间差异比较采用*t*检验和单因素方差分析法（*ANOVA*）分析ELISA、细胞流式术、各组间小鼠肿瘤体积变化，*P* < 0.05为差异有统计学意义。

## 2 结果

### 2.1 ELISA法检测细胞因子TNF-α、IFN-γ分泌结果

在T肽组和T肽联合顺铂组中，U937细胞培养上清液中TNF-α及IFN-γ浓度明显高于对照组和顺铂组，且各组间差异具有统计学意义。T肽联合顺铂组、T肽组*vs*对照组、顺铂组TNF-α：[72 h: (54.10±3.98) μmol/L, (28.70±5.19) μmol/L *vs* (3.19±1.13) μmol/L, (1.83±0.74) μmol/L, *P* < 0.05]；IFN-γ[72 h: (147.23±8.79) μmol/L, (66.75±5.74) μmol/L *vs* (2.54±0.99) μmol/L, (1.90±0.74) μmol/L, *P* < 0.05]。实验结果表明T肽具有促进巨噬细胞分泌肿瘤杀伤因子TNF-α、IFN-γ的作用，而且表明T肽能够减弱顺铂对巨噬细胞分泌细胞因子的抑制作用（图 1）。

**1:**
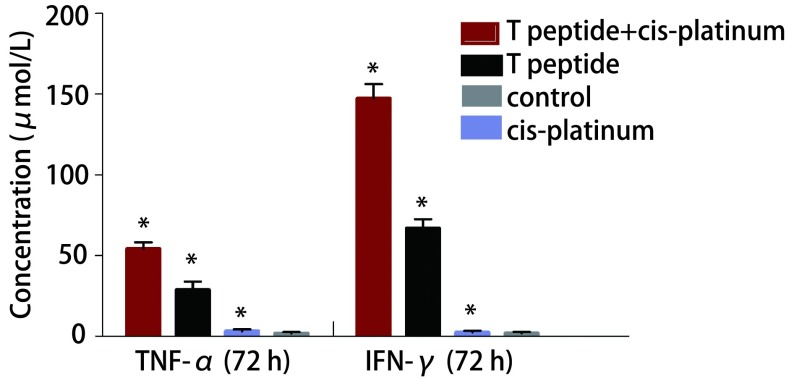
T肽对U937细胞分泌细胞因子TNF-*α*和IFN-*γ*的作用研究。不同处理组在72 h后，细胞培养上清液中TNF-*α*、IFN-*γ*浓度的变化情况。^*^P < 0.05 The concentration of TNF-*α* and IFN-*γ* of U937 cells treated with T piptide and/or cis-platinum after 72 h. ^*^P < 0.05. TNF-*α*: tumor necrosis factor-*α*; IFN-*γ*: interferon-*γ*.

### 2.2 用药后小鼠体重及移植瘤生长结果

结果显示T肽联合顺铂组对人鳞癌移植瘤生长抑制率优于顺铂组和T肽组（分别为92% *vs* 84%，*P* < 0.05；92% *vs* 48%，*P* < 0.05）（表 1）。此外，本实验期间顺铂组有3只小鼠死亡，其余组未见急性中毒反应。本实验通过记录实验期间各组小鼠体重描绘出体重变化曲线，从曲线中可以观察到T肽联合顺铂组、T肽组与对照组体重在实验期间变化不大，而单纯顺铂组小鼠体重呈现下降趋势（图 2，图 3）。

**1:** T肽在小鼠移植瘤模型中的肿瘤抑制作用 Anti-tumor activity of T peptide in exnograft mice model

Group	Route	Survival rate	Weight before treatment	Weight after treatment	Tumor weight	Tumor volume	Tumor inhibition rate
A	iH	100%	16.99±0.13	19.06±0.11	0.93±0.18	340.20±62	48%
B	iH/ip	100%	16.86±0.06	19.00±0.08	1.79±0.82	1, 620.93±64.6	-
C	iH/ip	100%	17.68±0.09	19.02±0.04	0.14±0.05	129.60±55.21	92%
D	ip	70%	17.74±0.04	15.32±0.02	0.28±0.03	263.28±48.21	84%
Group A: T peptide; Group B: control (0.9%NaCl); Group C: TP+cis-platinum; Group D: cis-platinum.							

**2:**
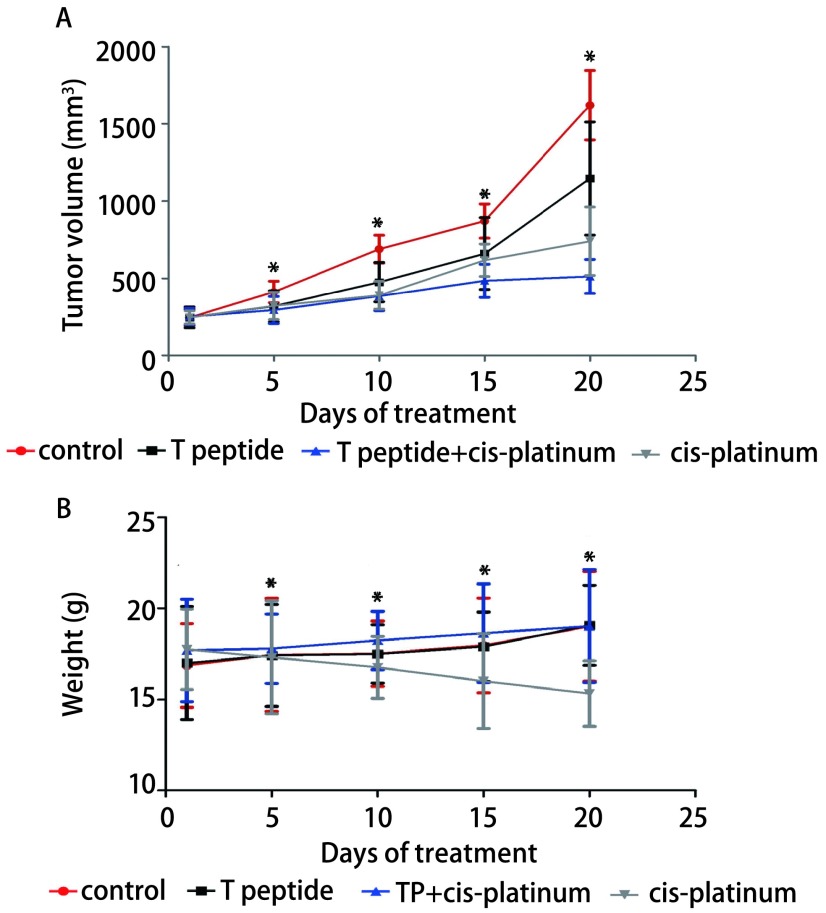
荷瘤小鼠模型以T肽和/或顺铂处理期间肿瘤体积及小鼠体重变化。A：小鼠肿瘤体积变化；B：用药期间小鼠体重变化情况。^*^P < 0.05。 The tumor volume and weight of exnograft mice when treated with T peptide and/or cis-platinum. A: The tumor volume were measured during the treatment. B: The mice weight were measured during the treatment. ^*^P < 0.05.

**3:**
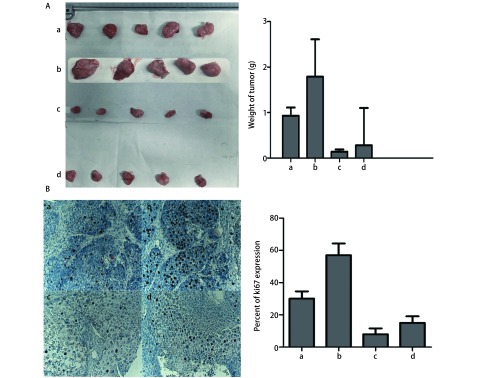
移植瘤小鼠模型用药后各组瘤块重量及ki67表达情况。A：荷瘤小鼠模型试验结束各组瘤块重量；B：各组瘤块免疫组化Ki-67染色表达率. a：T peptide；b：control；c：T peptide +cis-platinum；d：cisplatinum；^*^P < 0.05。 The tumor weight and Ki67 expression rate of Exnograft mice. A: The mice were sacrificed and tumors were removed and weighted; B: Immunohistochemistry assay for detectingthe proliferation marker Ki-67 in xenograft tumor sections. Representative microscopic images showing Ki-67-positive cells in each group. a: T peptide; b: control; c: T peptide+cis-platinum; d: cisplatinum. ^*^P < 0.05.

### 2.3 Ki67免疫组化结果

试验结束时，取出小鼠体内肿瘤进一步应用免疫组化分析发现，各组中肿瘤组织Ki67评分分别为T肽组*vs* Control组*vs* T肽+顺铂组*vs*顺铂组（++, +++, +, +）（图 3）。

### 2.4 细胞流式结果

实验收集用药期间小鼠尾静脉血观察T肽在小鼠移植瘤模型中对外周血巨噬细胞数量的影响。结果显示联合用药组小鼠尾静脉血中巨噬细胞比例明显增高。T肽联合顺铂组*vs* T肽组*vs*顺铂组*vs*对照组Day 1：[(2.24%±0.96%) *vs* (2.31%+0.77%) *vs* (2.29%±0.63%)*vs* (2.32%±0.51%)]、Day 15：[(11.69%±1.10%) *vs* (7.19%±0.99%) *vs* (0.62%±0.13%) *vs* (2.43%±0.77%)]（*P* < 0.05）（图 4）。

**4:**
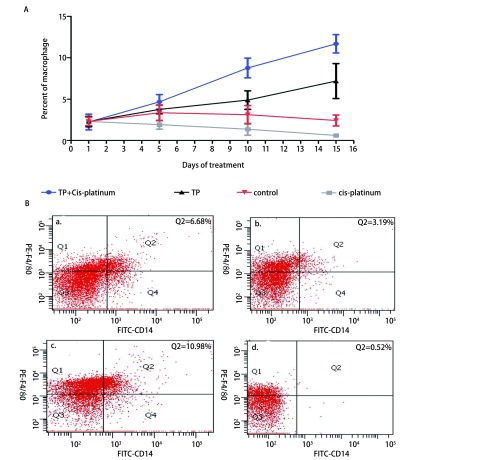
荷瘤小鼠模型以T肽和/或顺铂处理，外周血巨噬细胞的变化。A：用药期间各组小鼠外周血巨噬细胞变化曲线；B：用药周期结束时各组小鼠中随机抽取一只小鼠外周血巨噬细胞流式图。a：T peptide；b：control；c：T peptide+cis-platinum；d：cisplatinum。^*^P < 0.05。 The percentages of in were measured by FACS. A: The curve of exnograft mice peripheral blood macrophages during medication. B: Randomly selected flow cytometry of each group at the end of medication. a: T peptide; b: control; c: T peptide +cis-platinum; d: cisplatinum. ^*^P < 0.05.

## 3 讨论

T肽是Tuftsin的衍生物，Tuftsin是机体脾脏生成的一段生理活性肽，其基本功能是刺激巨噬细胞分泌细胞因子和巨噬细胞增殖。Tuftsin不能直接杀伤肿瘤细胞，但可以通过提高机体自身免疫力，增强效应细胞细胞毒性间接加强对肿瘤杀伤效应。早期研究认为肿瘤抗原的加工呈递主要由树突状细胞介导，巨噬细胞主要加工呈递外源性病原体抗原。2012年，Barrio等^[8]^通过研究黑色素瘤细胞发现巨噬细胞能够加工呈递黑色素瘤特异性抗原给T细胞从而激发机体特异性抗肿瘤免疫应答。巨噬细胞杀伤肿瘤细胞主要是通过抗原呈递和分泌具有肿瘤杀伤作用的细胞因子。巨噬细胞通过肿瘤抗原呈递激活上调T细胞和B细胞的MHC-II表达，导致CD4+T细胞反应性提高，从而促进诱导T细胞的增生以及活化细胞毒性T淋巴细胞，杀灭肿瘤细胞。在II期临床试验中发现Tuftsin单独用于早期肿瘤患者治疗效果并不理想^[9]^，但与化疗药物联合使用对小细胞肺癌的治疗效果非常显著^[10]^。由于其生物活性低，在体内易于水解失效，在此基础上合成其衍生物T肽，T肽具有更高的生物活性。T肽拥有4个Tuftsin样结构，体内药物代谢研究表明，T肽在体内的半衰期约2 h-4 h，与Tuftsin在体内2.8 h^[11]^的半衰期相比，明显增强了生物活性。

T肽基本功能与Tuftsin相似，主要效应细胞是巨噬细胞。在抗肿瘤免疫调节中巨噬细胞具有重要的地位，可直接或者通过呈递肿瘤相关抗原诱导机体免疫应答杀伤肿瘤细胞。目前肿瘤间质中存在肿瘤相关巨噬细胞（tumor associated macrophages, TAM）在肿瘤微环境的调控下可以变换表型，演变为抑制肿瘤生长的M1型或促进肿瘤生长的M2型^[12, 13]^。相关研究^[14]^表明T肽可以结合巨噬细胞表面的特异性抗体，激活NF-қB信号通路，增强巨噬细胞的吞噬功能同时介导巨噬细胞向M1型巨噬细胞转化，促进Th1相关的细胞因子，如TNF-α、IL-12、IFN-γ等。在巨噬细胞分泌细胞因子中TNF-α、IFN-γ是巨噬细胞分泌的具有抗肿瘤效果的主要细胞因子，TNF-α是一种具有多种生物活性的细胞因子，可引起肿瘤细胞凋亡^[15]^，只有活化的巨噬细胞才具有分泌细胞因子的作用。已有研究证实T肽具有激活巨噬细胞并促进巨噬细胞分泌细胞因子，本实验也证实T肽联合顺铂后细胞因子TNF-α、IFN-γ分泌明显高于其他三组。

在小鼠移植瘤实验结果显示T肽与顺铂联合用药组肿瘤抑制作用优于其他三组的同时，小鼠一般情况好，未见顺铂引起的严重毒性反应。用药期间流式检测结果提示联合用药组小鼠外周血中活化巨噬细胞明显高于顺铂单药组。对比顺铂单药组与联合用药组，联合用药组对巨噬细胞的活化作用明显强于顺铂单药组，这可能与T肽刺激单核细胞向巨噬细胞分化并活化巨噬细胞有关。T肽作为化疗辅助用药，不仅能提高化疗对肿瘤细胞的杀伤作用的同时还可以增强免疫功能减少化疗对正常免疫细胞的毒副作用。

在临床肿瘤治疗中，通常采用手术切除肿瘤后辅助放化疗。但是手术仅解决肉眼可见的实体瘤，一些微小病灶或手术操作中掉落的肿瘤细胞并无法通过手术根除。由于肿瘤患者一般状态较差并且手术对机体造成二次创伤，大部分术后短期内无法使用化疗药物。因此防止术后肿瘤转移复发成为肿瘤术后的难题。在术后与第一次化疗治疗期间的空窗期为肿瘤二次生长及远处转移创造机会。因为T肽具有调节机体免疫状态，通过巨噬细胞增强机体对肿瘤细胞的杀伤作用。介于T肽具有通过调节自身免疫间接杀伤肿瘤细胞，我们认为可能早期在术后使用T肽可以减少术后肿瘤细胞生长及远处转移的风险，患者是否可以获益仍需要更多临床试验验证。

本实验结果显示，T肽能够促进巨噬细胞增殖和提高其分泌肿瘤细胞杀伤因子（TNF-α、IFN-γ）；在体外动物模型中，T肽能够与化疗药物产生协同作用，提高化疗药物如顺铂的疗效，同时能够减轻化疗药物的毒副作用。
